# Climate-induced changes in carbon flows across the plant-consumer interface in a small subarctic lake

**DOI:** 10.1038/s41598-019-53541-3

**Published:** 2019-11-19

**Authors:** Simon Belle, Jenny L. Nilsson, Ilmar Tõnno, Rene Freiberg, Tobias Vrede, Willem Goedkoop

**Affiliations:** 1 Department of Aquatic Sciences and Assessment, Swedish University of Agricultural Sciences, Uppsala, Sweden; 2 Centre for Limnology, Institute of Agricultural and Environmental Sciences, Estonian University of Life Sciences, Tartu County, Estonia

**Keywords:** Boreal ecology, Palaeoecology, Limnology

## Abstract

Reconstructions of past food web dynamics are necessary for better understanding long-term impacts of climate change on subarctic lakes. We studied elemental and stable isotopic composition of sedimentary organic matter, photosynthetic pigments and carbon stable isotopic composition of *Daphnia* (Cladocera; Crustacea) resting eggs (δ^13^C_Clado_) in a sediment record from a small subarctic lake. We examined how regional climate and landscape changes over the last 5800 years affected the relative importance of allochthonous and autochthonous carbon transfer to zooplankton. Overall, δ^13^C_Clado_ values were well in line with the range of theoretical values of aquatic primary producers, confirming that zooplankton consumers in subarctic lakes, even in the long-term perspective, are mainly fuelled by autochthonous primary production. Results also revealed greater incorporations of benthic algae into zooplankton biomass in periods that had a warmer and drier climate and clearer water, whereas a colder and wetter climate and lower water transparency induced higher contributions of planktonic algae to *Daphnia* biomass. This study thus emphasizes long-term influence of terrestrial-aquatic linkages and in-lake processes on the functioning of subarctic lake food webs.

## Introduction

Arctic/subarctic ecosystems are exposed to rapid and extensive changes driven by accelerated warming^1^, including changes in vegetation cover, biomass and productivity (i.e. the greening of the Arctic^2^;), and permafrost thaw^3^. These climate-driven landscape transformations dramatically affect run-off patterns, biogeochemical cycles and organic matter dynamics, potentially triggering cascading effects acting at regional and global scales^4,5^. Arctic/subarctic landscapes are also characterized by a high density of lakes and water courses, exceeding 4% of the total land area^6^. Direct effects of warming on lakes affect both their physical (e.g. timing and duration of ice cover^7^), chemical (e.g. changes in organic matter inputs and nutrients run-off ^8,9^) and biological (e.g. community composition^10^) properties. In northern landscapes, surface waters also play a key role in the transport of detrital materials, nutrients and organic matter^11,12^, thus indirectly determining the relative role of allochthonous (terrestrial) and autochthonous food resources available for aquatic consumers.

Energy flows through aquatic food webs are of fundamental importance for the function of lake ecosystems^13^, and are a key to our understanding of their response to global change. Biological communities in arctic/subarctic lakes are often species-poor and have simpler food webs than those of temperate lakes^14^. Carbon transfer through food webs is derived either from autochthonous primary production (i.e., phytoplankton and benthic algae^15,16^,), from allochthonous detrital inputs and/or from remobilized dissolved organic carbon by bacteria^17^. However, due to the lack of long-term contemporary data of subarctic lakes, it is still unclear to what extent climate change affects the relative importance of these trophic processes. Hence, past dynamics of aquatic ecosystems from paleo-data provide key information that can contribute to our understanding of lake responses to changes^18^.

Recent paleolimnological studies conducted on subarctic lakes have shown complex relationships between changes in terrestrial vegetation in the watershed, inputs of allochthonous organic matter, changes in in-lake light regime and taxonomic shifts in the assemblages of aquatic primary producers^19–21^. However, identification of past energy pathways within subarctic lake food webs has been less extensively studied^21^. Innovative approaches using stable isotope composition (mainly carbon, nitrogen and hydrogen) of invertebrate remains archived in lake sediment records have recently been developed to reconstruct past energy flows to aquatic primary consumers^22–24^. Specifically, Cladocera (Crustacea) form an important trophic link between basal resources (i.e., primary producers, bacteria) and fish, and stable isotopic composition of their sclerotized remains (e.g. resting eggs or ephippium) is thus a good indicator of long-term changes in aquatic food webs^25^. These paleolimnological approaches help to provide insight in future trajectories of lake food webs under ongoing climate and land-cover change in Arctic and subarctic regions.

The main objective of this study was to investigate the past relationships between climate change, landscape development and carbon flows to zooplankton in a subarctic lake. We analysed sediment composition and photosynthetic pigments, as well as carbon stable isotope composition of *Daphnia* (Cladocera; Crustacea) resting eggs to reconstruct past dynamics of sedimentary organic matter, autochthonous primary production and carbon resources available to Cladocera under different climatic conditions. Results were then compared with those of previous paleolimnological studies that focused on climate and landscape changes to estimate the relative importance of in-lake impacts *vs*. catchment-mediated processes on energy flows through subarctic food webs.

## Methods

### Study site

**Figure 1:**
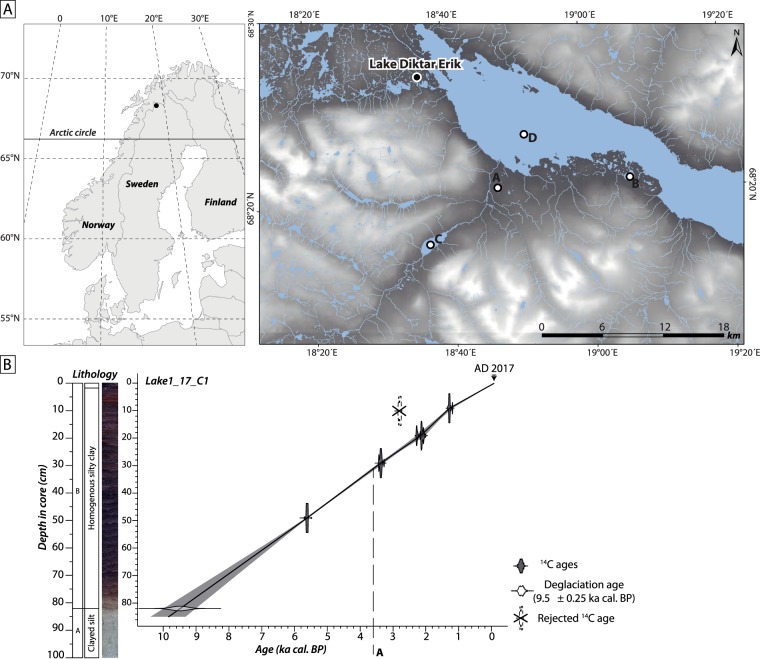
(**A**; left) Location of the study region showing the sampling site (black circle; Lake Diktar Erik). (right) Elevation map over the study region showing the major lakes, streams and rivers. Black circle marks the sampling site (Lake Diktar Erik), whereas open ones indicate locations of selected paleolimnological studies from the area (**A**: Vuolep Njakajaure, **B**: Vuoskkujávri, **C**: Abiskojaure and **D**: Torneträsk). (**B**) Picture, lithological description and age–depth model (linear interpolation) of the sediment core retrieved from Lake Diktar Erik. Letter “A” refers to major changes reported in sediment, pigment and cladoceran data (see Fig. 5).

Lake Diktar Erik (68°26′43″N, 18°36′50″E) is a small lake (0.1 km^2^) located in northern Sweden (Fig. 1A). The bedrock geology in the region is predominated by granite and its metamorphic products, while the prevailing catchment vegetation consists of mountain birch forest (*Betula pubescens* ssp. *tortuosa*). The lake is located at 375 m a.s.l. and has a maximum water depth of 16 m (Secchi depth of 6 m). The lake is oligotrophic and slightly humic, with a pH of 6.3, conductivity of 14.7 µS.cm^−1^, and concentrations of total organic carbon of 3.6 mg.L^−1^, total phosphorus (TP) of 5 µg P L^−1^ and total nitrogen (TN) of 206 µg N L^−1^ recorded in 1997^26^. Similar concentrations of DOC: 4.2 mg L^−1^, total phosphorus: 4 µg TP L^−1^ and total nitrogen: 270 µg TN L^−1^ for this lake were also reported by Karlsson *et al*.^27^.

Deglaciation of the surroundings of Lake Diktar Erik was reported at approximately 9.5 ka cal. BP^28–30^. Then, favourable climatic conditions allowed rapid transition from a vegetation-free landscape to forest vegetation during the early Holocene (*ca*. 9.5–6.5 ka cal. BP)^29–31^. This initial phase of landscape development was followed by a climatically stable and warm period during the Holocene Thermal Maximum (*ca*. 6.5–3.5 ka cal. BP), marked by widespread developments of pine forests. The late Holocene period (*ca*. 3.5–0.05 ka cal. BP) was instead characterised by a long-term marked cooling and wetter conditions inducing a gradual replacement of pine forest by a mountain birch forest similar to that present nowadays^30–32^. Previous paleoecological studies from the area have not identified distinguishable environmental impacts in response to early human activities^33^. During the 20^th^ century, rail tracks and road constructions have occurred along the southern, downstream shore of the lake. Thus, Holocene development of northern Sweden provides an exceptional natural experiment to study how climate and landscape changes affected terrestrial-aquatic linkages and carbon flows across the plant-consumer interface in subarctic lakes.

### Sediment sampling

In August 2017, a 100-cm sediment core was retrieved from the largest depth of the lake using a gravity corer (9 cm of diameter; UWITEC). As terrestrial macrofossils were absent in the collected core, radiocarbon dates were determined on five bulk sediment samples measured using an accelerator mass spectrometer at the Poznan Radiocarbon Laboratory (Poland) and The Tandem Laboratory (Sweden; Table 1). In addition, we constrained the marked transition between proglacial and lacustrine sediments (Fig. 1B) using the deglaciation age (*ca*. 9.5 ± 0.25 ka cal. BP) reported by previous paleolimnological studies conducted in the region^34,35^. Age-depth modelling, combining calibrated radiocarbon dates and deglaciation age, was performed using simple linear interpolation (Clam package for R^36^;).

**Table 1:** Radiocarbon dates (±1 standard deviation) of the sediment sequence (Lake1_17_C1) from Lake Diktar Erik. In column Model, “R” refers to rejected date, and “I” to included dates.

Depth (cm)	Lab code	Material	yr BP	Model
9	Ua-62416	Bulk	1356 ± 31	I
10	Poz-99924	Bulk	2685 ± 30	R
19	Ua-62417	Bulk	2147 ± 31	I
29	Poz-99925	Bulk	3145 ± 30	I
49	Poz-99926	Bulk	4885 ± 35	I

### Sedimentological and sedimentary pigment analysis

The sediment core was vertically split in two halves, and one split core surface was covered with Ultralene® foil to avoid desiccation and contamination, and scanned every 3 mm using an ITRAX XRF Core Scanner at the University of Stockholm (Cox Analytical Systems). XRF measurements were carried out using a Mo tube, set at 30 kV and 30 mA, for 60 s to detect relative concentrations of selected major elements (Ti, Fe, Mn, Si, Al). Elemental intensities were expressed as counts per unit time per unit area (cps).

The other split core was continuously and horizontally sliced down to 50 cm depth into 1 cm thick sub-samples that were subjected to further analyses. Organic matter concentration (OM) was analysed using the loss-on-ignition method, and results were expressed as percentage of dry weight (hereafter; % of dry weight). In addition, sediment samples were analysed for carbon and nitrogen stable isotopes (depicted as δ^13^C_OM_ and δ^15^N_OM_, respectively), carbon and nitrogen concentrations (C_org_ and N_tot_), and C/N weight ratios. Prior to analysis, sediment samples were dried (at 60 °C for 72 h), ground, and 3 mg of dried sediments were transferred to tin capsules. δ^13^C_OM_ and δ^15^N_OM_ were analysed using an Isotope Ratio Mass Spectrometer interfaced with an Elemental Analyser (EA-IRMS) at our Stable Isotope Laboratory (Umeå, Sweden). Results were expressed as the delta notation with Vienna Pee Dee Belemnite and atmospheric nitrogen as standards: δ^13^C or δ^15^N (‰) = [(R_sample_/R_standard_) − 1] ×1000; where R = ^13^C/^12^C or ^15^N/^14^N. Sample measurement replications from internal standards (wheat and maize flour) produced analytical errors (1σ) of ±0.15‰ for both δ^13^C and δ^15^N values (*n* = 24).

Photosynthetic sedimentary pigments were analysed as a paleo-proxy of aquatic primary production^20^, following the method by^37^. Briefly, sediment samples were freeze-dried, and pigments were extracted at −20 °C in the dark during 24 h using a solution of acetone and methanol (80:20 V:V). Extracts were then clarified by filtration through a 0.45 µm Millex-LCR hydrophilic PTFE membrane filter before chromatographic analysis. Reversed-phase high–performance liquid chromatography (RP-HPLC) was applied to separate pigments. A Shimadzu Prominence (Japan) series binary gradient system with a photodiode array (PDA) and fluorescence detectors was used (see^38^ for details). Peak identification and quantification were made by commercially available external standards from DHI Company (Denmark). Chlorophyll *a* (Chl *a*) and its derivative pheophytin *a* (Phe *a*) were selected to indicate the overall algal biomass^37,39^. The Chl *a*/ Phe *a* ratio was used to assess pigment preservation in lake sediments, and ratios are expected to remain relatively stable over time if stabile preservation conditions occur^40^. Taxon-specific pigments were used to indicate the biomass of aquatic primary producer’s classes: lutein (Lut) for green algae (Chlorophyceae), fucoxanthin (Fuco) for diatoms (Bacillariophyceae), alloxanthin (Allo) for cryptophytes (Cryptophyteae^39^,) and canthaxanthin (Cantha) for cyanobacteria (Cyanophyceae^41^,). Pigment concentrations are expressed as nanomoles per gram of sediment organic matter (nmol g^−1^ OM).

### Carbon stable isotope analysis of cladoceran remains

Stable isotope analyses were performed on resting eggs of *Daphnia* ssp. morphotype retained from lake sediment layers and identified using the photograph book of Szeroczyńska and Sarmaja-Korjonen^42^. Sediment samples were deflocculated in NaOH (10%) solutions, pre-treated using washing with HCl (10%) solutions and sieved through a 100-µm mesh according to standard protocol of Perga^22^. Resting eggs were sorted out under a dissection microscope until approximately 50 eggs or a mass of about 60 µg (minimal mass required for stable isotope analysis) was gained. If resting egg abundances in a single sediment layer were too low, then the next consecutive sediment layer was pooled to the sample. Carbon stable isotopic composition of cladoceran remains (δ^13^C_Clado_) was then analysed using an EA-IRMS at INRA Nancy (Champenoux) expressed according to the delta notation (see above). Replication of sample measurements from internal laboratory standards produced analytical errors (1σ) of ±0.2‰ (*n* = 15).

### Data analysis

Two separate principal component analyses (PCA) were performed on sediment and pigment data, respectively. PCA axis significance was checked using the broken-stick model^43^. Pigment concentrations and sediment composition were expected to have significant, and potentially non-linear, influences on food resources for zooplankton and, therefore, δ^13^C_Clado_ values. Statistical relationships between δ^13^C_Clado_ values and PCA1 scores performed on sedimentological and pigment data (PCA1_sed_ and PCA1_pig_, respectively) were examined using a generalized additive model (GAM; fitted using the *mgcv* package for R^44^;) approach, with a continuous-time, first-order autoregressive process to account for temporal autocorrelation^45^. Significance of fitted trends was checked using standard statistical inferences for GAM. All statistical analyses and plots were performed using the R 3.5.2 software^46^.

## Results

### Past changes in sediment composition

**Figure 2:**
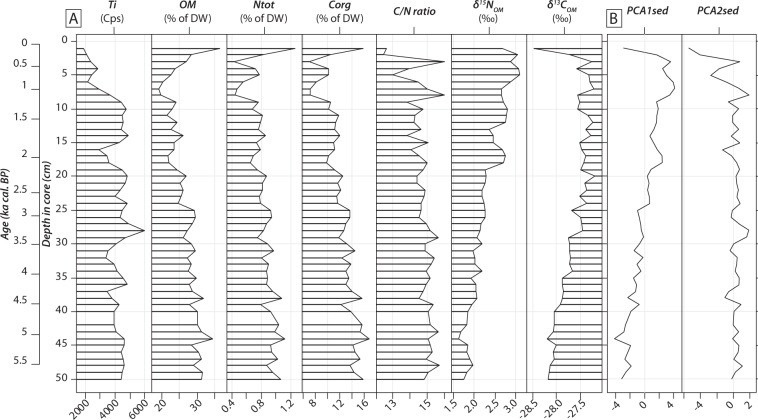
(**A**) Trends in titanium intensities in lake sediments (Ti; cps), organic matter concentration (OM; % of dry weight), total nitrogen (N_tot_; % of dry weight), total organic carbon (C_org_; % of dry weight), atomic ratio of organic carbon to total nitrogen (C/N), stable nitrogen isotopic composition of sedimentary organic matter (δ^15^N_OM_; ‰), and stable carbon isotopic composition of sedimentary organic matter (δ^13^C_OM_; ‰). (**B**) Temporal trends in scores of first and second principal component analysis axes (PCA1_sed_ and PCA2_sed_) performed on sediment data. Age and depth scales are both given on y-axes.

The transition from proglacial to lacustrine sedimentation could be well observed in our sediment core as a colour-change from dark grey at the bottom to dark brown towards the surface (Fig. 1B). All calibrated radiocarbon ages consistently increase with depth in core, except at 10 cm (Table 1), and this radiocarbon age was therefore not included in the final model as it would lead to age-reversal and/or abrupt change in sedimentation rates not supported by sedimentological observations (Fig. 1B). This radiocarbon age also conflicted with another date at 9 cm depth (Table 1) which fits well the age model. Hence, 81 cm of sediments from Lake Diktar Erik covered the last *ca*. 9500 years, corresponding to an average sedimentation rate of about 0.08 mm.yr^−1^. Titanium intensities in lake sediments were relatively stable at approximately 4500 cps from 50 to 10 cm sediment depth, and then gradually decreased to 2000 cps at the sediment surface (Fig. 2A). Organic matter concentration (OM) ranged 19.1–36.5%, while C_org_ and N_tot_ concentrations in sediments ranged 6.9–16.9% and 0.5–1.2%, respectively (Fig. 2A). Overall, OM, C_org_ and N_tot_ concentrations were higher in the oldest part of the record (*ca*. 5.8–3.5 ka cal. BP), and followed a gradual decrease over time (from *ca*. 3.5 to 0.05 ka cal. BP). Interestingly, however, these declining trends reversed and showed conspicuous peaks in the most recent sediment layers. The sediment weight C/N ratio ranged 12.5–16.0 with the highest values observed from *ca*. 5.8 to 3.5 ka cal. BP (Fig. 2A). δ^15^N_OM_ values ranged 1.6–3.1‰, and those of δ^13^C_OM_ ranged from −28.5 to −27.2‰. δ^13^C_OM_ and δ^15^N_OM_ temporal trends were similar, with gradual increases over time, except for the uppermost samples where instead decreases were noted (Fig. 2A).

**Figure 3:**
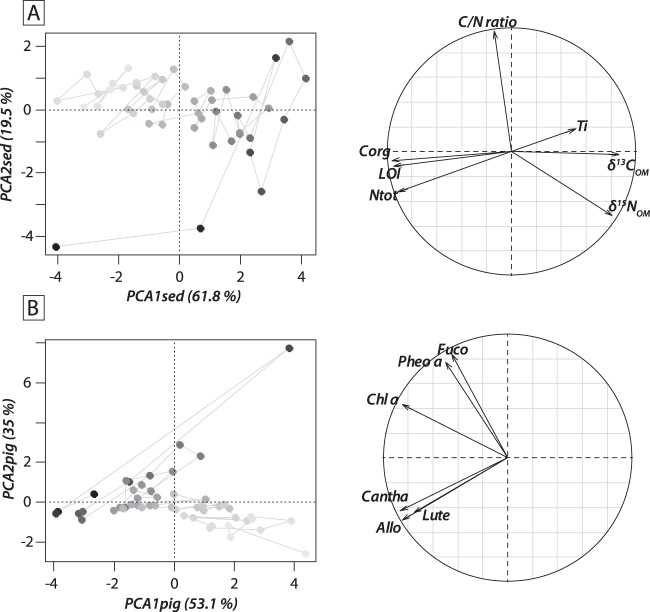
Factorial map of principal component analyses (PCA1 *vs*. PCA2) performed on (**A**) sedimentological data and (**B**) individual sedimentary pigment data. A grey-scale was used to identify the sample age: light-grey colours correspond to the oldest samples, whereas black symbols represent the youngest samples. Correlation circles representing variable contributions to the first two axes of the PCA.

The first two PCA axes explained 61.8% and 19.5%, respectively, of the total variance of sediment data. PCA1_sed_ axis was strongly correlated with OM, N_tot_, C_org_, δ^15^N_OM_ and δ^13^C_OM_ (Fig. 3A). Negative values on PCA1_sed_ axis represented organic-rich sediment layers with low δ^15^N_OM_ and δ^13^C_OM_ values. PCA2_sed_ axis predominantly explained C/N ratios (Fig. 3A), with negative values representing samples with low C/N ratios. PCA1_sed_ scores followed a gradual increase over time, switching from negative to positive values at *ca*. 3.4 ka cal. BP (Fig. 2B), whereas PCA2_sed_ scores showed no specific temporal trend, except a conspicuous decrease observed from 8 cm and upward (Fig. 2B).

### Trends of sedimentary pigment concentrations

**Figure 4:**
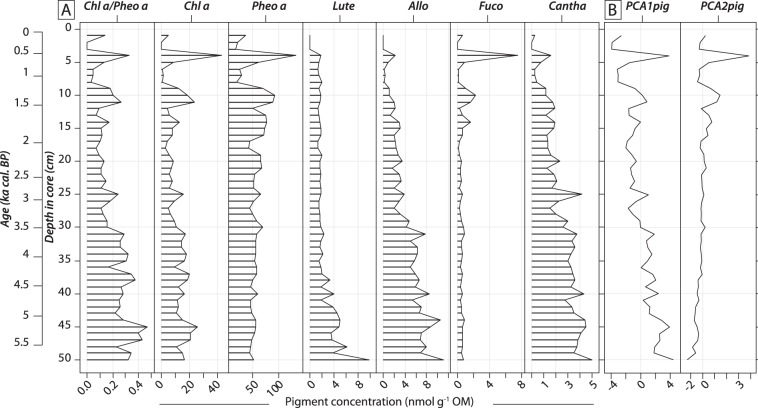
(**A**) The stratigraphic diagram shows temporal trends of investigated sedimentary pigments. Individual pigments are expressed in terms of nanomoles per gram of organic matter (nmol g^−1^ OM). Pigment names are abbreviated as follows (from left to right): chlorophyll *a*: Chl *a*, pheophytin *a*: Pheo *a*; lutein: Lut, alloxanthin: Allo, fucoxanthin: Fuco, canthaxanthin: Cantha. (**B**) Temporal trends in scores of first and second principal component analysis axes (PCA1_pig_ and PCA2_pig_) performed on sedimentary pigment data. Age and depth scales are both given on y-axes.

Chl *a*/Phe *a* ratios showed high values in deep sediment layers, but gradually declined from 50 to 13 cm depth in the core (Fig. 4A). The highest photosynthetic pigment concentrations, 132.0 nmol g^−1^ OM and 43.1 nmol g^−1^ OM, respectively, were found for Phe *a* and Chl *a* (Fig. 3A). Allo and Lute, indicating cryptophytes and green algae, respectively, were the most concentrated taxon-specific pigments (ranging 2–9 nmol g^−1^ OM, Fig. 4A). Temporal trends of pigments were almost consistently similar, higher during the oldest part of the record (*ca*. 5.8–3.5 ka cal. BP), and then decreasing gradually to present day. However, Fuco instead showed slightly higher values from *ca*. 3 ka cal. BP to present-day, indicating an increase in diatoms. The first two axes of the PCA applied to sedimentary pigment data accounted for 53.1% and 35.0% of the total variance, respectively. PCA1_pig_ axis explained Chl *a*, Allo, Cantha and Lute (Fig. 3B). Negative values on the PCA1_pig_ axis represent pigment-rich sediment layers. PCA2_pig_ axis predominantly explained Pheo *a* and Fuco (Fig. 3B), with positive values representing sediment samples with high Fuco and Pheo *a* concentrations. PCA1_pig_ scores followed a gradual decrease over time, switching from positive to negative values around 3.5 ka cal. BP (Fig. 4B) suggesting a decline in concentrations of Chl *a*, Allo, Cantha and Lute, whereas PCA2_pig_ scores followed a small increase over time (Fig. 4B).

### Stable C isotope in cladoceran resting eggs

**Figure 5:**
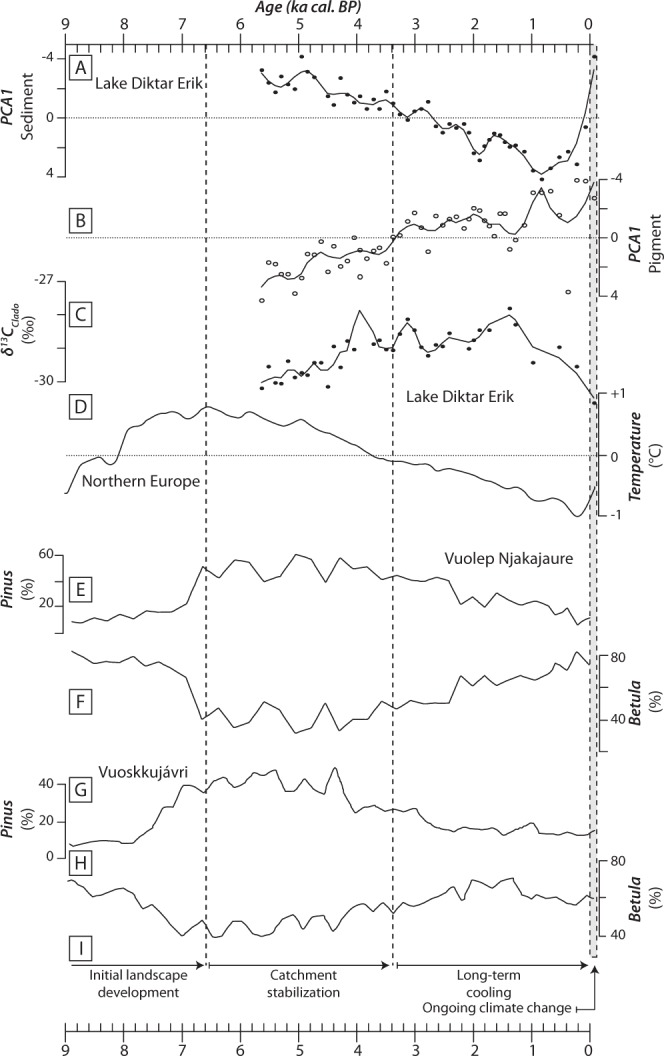
(**A**) Sediment PCA1 scores, (**B**) pigment PCA1 scores, (**C**) carbon stable isotopic composition of cladoceran remains in sediment core Lake1_17_C1 from Lake Diktar Erik (δ^13^C_Clado_; ‰), (**D**) pollen-based temperature variability for Northern Europe^47^, percentage of pollen from (**E**) *Pinus sylvestris* and (**F**) *Betula pubescens* in sediment core of Vuolep Njakajaure (letter A in Fig. 1^30^), percentage of pollen from (**G**) *Pinus sylvestris* and (**H**) *Betula pubescens* in sediment core of Vuoskkujávri (letter B in Fig. 1^31^), and (**I**) vertical dashed lines dividing the stratigraphy into 4 phases: initial landscape development (*ca*. 9.5–6.6 ka cal. BP), catchment stabilization (*ca*. 6.6–3.4 ka cal. BP), long-term cooling (*ca*. 3.4–0 ka cal. BP) and ongoing climate change (adapted from^28^).

**Figure 6:**
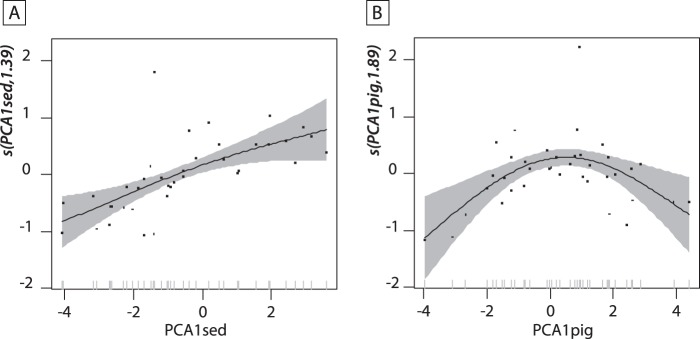
Fitted smooth function between explanatory variables (**A**: PCA1_sed_ and **B**: PCA1_pig_) and δ^13^C_Clado_ values from a generalized additive model (GAM), with a continuous-time first-order autoregressive process to account for temporal autocorrelation. Grey surface marks the 95% uncertainty interval of the fitted function. On the x-axis, black ticks show the distribution of observed values for variables. Numbers in brackets on the y-axis are the effective degrees of freedom (*edf*) of the smooth function.

δ^13^C values of *Daphnia* resting eggs ranged from −30.6 to −27‰ (Fig. 5C), and the lowest δ^13^C_Clado_ value was found for the uppermost sediment layer (−30.6‰; Fig. 5C). Based on the temporal trends, two distinct patterns were identified in the uppermost 50 cm of the core. The δ^13^C_Clado_ values first increased from −30.6 to −28.8‰ between 5.8 and *ca*. 3 ka cal. BP, and then decreased to values around −30.2‰ after *ca*. 3 ka cal. BP. GAM showed that PCA1_sed_ and PCA1_pig_ covariates explained 56.9% of the overall variability of δ^13^C_Clado_ values (*p*-value < 0.001). Relationships between δ^13^C_Clado_ values and covariates were non-linear (Fig. 6). PCA1_sed_ showed a monotonic and positive relationship with δ^13^C_Clado_ values (F = 7.2; *edf* = 1.39; Fig. 6A), whereas PCA1_pig_ scores were unimodally related to them (F = 7.9; *edf* = 1.89; Fig. 6B). The PCA1_pig_ fitted function showed a positive relationship with δ^13^C_Clado_ values for negative PCA1_pig_ scores, and a negative relationship with δ^13^C_Clado_ values for positive PCA1_pig_ scores. Therefore, major shift in pigment response curve occurred for PCA1_pig_ scores observed at *ca*. 3.5 ka cal. BP (Figs. 5 and 6).

## Discussion

We reconstructed the long-term development of Lake Diktar Erik over the last 5800 years based on sedimentary organic matter and photosynthetic pigment analyses. Our results showed that during the Holocene Thermal Maximum (5.8–3.5 ka cal. BP), steady organic matter inputs from the surrounding watershed were revealed by high OM and C_org_ concentrations and high C/N ratios in Lake Diktar Erik sediment core (Fig. 2). Moreover, the highest pigment concentrations in the Lake Diktar Erik record were also observed during this period (Fig. 4), a period that otherwise showed a general decline in all pigment concentrations (Fig. 4). This decline in autochthonous primary production was likely driven by the onset of the cooling phase around 5 ka cal. BP in Fennoscandia (Fig. 5D^45^; but see also^21,28,29^). Then, the climate during the late Holocene period (*ca*. 3.5–0.05 ka cal. BP) was characterised by long-term cooling and wetter conditions (Fig. 5D)^47^, inducing a gradual replacement of pine forest by a mountain birch forest similar to that present nowadays (Fig. 5E–H)^30–32^. These results were well in line with successional developments of similar lakes in northern Sweden (see also^28,30,32^), and showed three distinct development phases. Our results also showed a strong decline in OM and pigment concentrations (Figs. 2 and 4), suggesting marked decreases in both terrestrial and aquatic productivity, as previously reported from pigment and diatom dynamics^21,29^ and sedimentological records from other subarctic lakes^30,31^. Moreover, the ongoing trend towards more oligotrophic conditions revealed from long-term monitoring of Swedish lakes^11^ corroborate this observation. Whereas the overall trend in PCA1_sed_ scores followed a gradual increase over time, switching from negative to positive values, the uppermost sediment sample instead showed the most negative PCA1_sed_ value of the time series (Fig. 5A), likely due to the effects of diagenetic alteration of both elemental and isotopic compositions of sedimentary organic matter (see also^48,49^).

The δ^13^C values of *Daphnia* resting eggs (δ^13^C_Clado_) ranged from −30.6 to −27‰, and were lower than those of organic matter (Fig. 5C), implying that zooplankton utilised an isotopically lighter food source than the overall sedimentary organic matter. δ^13^C_OM_ values have been widely used as a reliable proxy of sediment composition, reflecting the relative contribution of organic matter from aquatic and terrestrial origins, as autochthonous primary producers usually exhibit lower δ^13^C values than those of terrestrial organic matter^50^. Therefore, *Daphnia* of Lake Diktar Erik likely has preferentially assimilated ^13^C-depleted aquatic primary producers, and our study thus confirms that zooplankton consumers in subarctic lakes, even in the long-term perspective, have been mainly fuelled by autochthonous primary production (see also^51–54^). The δ^13^C_Clado_ values were also closely correlated to changes in sediment composition (PCA1_sed_ scores) and in-lake primary production (PCA1_pig_ scores), but we found a major change in their relationships with in-lake primary production occurring at *ca*. 3.5 ka cal. BP (Figs. 5B and 6B). This pattern suggest a strong influence of autochthonous primary production on *Daphnia* feeding habits, and these results could strengthen previous findings that zooplankton diet and algal dynamics are closely linked in subarctic food webs.

Based on long-term trends in Lake Diktar Erik and previous paleolimnological investigations of other lakes from the area, we identified the potential mechanisms of these observed patterns. Between 5.8 and *ca*. 3.5 ka cal. BP, δ^13^C_Clado_ values increased from −30.6 to −28.8‰, and were positively correlated to a decrease in autochthonous primary productivity (Fig. 6A). This increase in δ^13^C_Clado_ values could thus reflect a slight increase in the relative contribution of ^13^C-enriched terrestrial organic matter to *Daphnia* biomass. Our study thereby strengthens previous findings that the relative contribution of allochthonous organic matter to consumer biomass largely increased in unproductive lake food webs (Fig. 6^55,56^). After *ca*. 3.5 ka cal. BP, δ^13^C_Clado_ values decreased and correlated negatively with pigment data (Fig. 6B). Several studies of similar subarctic lakes during this period have demonstrated a taxonomic shift in algal assemblage composition (from benthic- *vs*. pelagic-dominated algal assemblages) induced by increased inputs of terrestrial DOC and a decline in the phototrophic zone of the lake^19,21^. Specifically, there is evidence that cooler and wetter climate conditions reported in Fennoscandia during the late Holocene induced a change in catchment vegetation, increased transport of DOC to lakes and a subsequent functional predominance of benthic to pelagic algae^19,21^. Our observed trends in sediment composition and pigment concentrations (Figs. 2 and 4) are typical for this type of lakes^19,21^, and we therefore conjecture that a similar shift from a benthic- to a pelagic-dominated algal assemblages occurred in Lake Diktar Erik during this period. As phytoplankton usually is more ^13^C-depleted than benthic algae^57,58^ and terrestrial organic matter^50^, an observed decrease in δ^13^C_Clado_ values would therefore indicate a higher contribution of planktonic algae to zooplankton biomass. Results suggested that the diet of *Daphnia* in subarctic lakes, even in the long-term perspective, depends on both availability (i.e. standing stock) and quality of food resources (i.e. allochthonous *vs*. autochthonous; benthic *vs*. pelagic), as previously demonstrated at seasonal scale (see also^59^). Our study thus revealed the long-term influence of terrestrial-aquatic linkages and in-lake processes on the functioning of subarctic food webs.

In this study, we examined how regional climate and landscape changes over the last 5800 years affected the relative importance of allochthonous and autochthonous carbon transfer to zooplankton in a subarctic lake. Our study revealed complex interplays between climate-induced change in in-lake (through algal productivity and assemblage composition) and catchment-mediated (through changes in allochthonous DOC and vegetation composition) processes in the functioning of planktonic food webs of a small subarctic lake. The results showed greater incorporations of benthic primary production into zooplankton biomass in periods that had a warmer and drier climate and clearer water, whereas colder and wetter climates and lower water transparency led to higher relative scontributions of planktonic algae into zooplankton biomass. Hence, our results demonstrate that paleolimnological studies can be a powerful approach for further exploring impacts of climate change on biogeochemical cycles and terrestrial-aquatic linkages in subarctic environments.
